# Bilateral Ocular Necrotizing Fasciitis in an Immunosuppressed Patient on Prescription Eye Drops

**DOI:** 10.7759/cureus.9129

**Published:** 2020-07-11

**Authors:** Kelly C Landeen, Paul W Mallory, Brian P Cervenka

**Affiliations:** 1 Otolaryngology - Head and Neck Surgery, Vanderbilt University Medical Center, Nashville, USA; 2 Ophthalmology and Visual Neurosciences, University of Minnesota, Minneapolis, USA; 3 Otolaryngology, University of Cincinnati College of Medicine, Cincinnati, USA

**Keywords:** necrotizing fasciitis, orbital cellulitis, sinus disease, immunosuppression, eye drops, rheumatoid arthritis

## Abstract

Preseptal cellulitis is an infection of ocular tissue that is often unilateral and caused by extension of sinonasal disease. In rare instances it can lead to life-threatening necrotizing fasciitis. We present here a unique case of bilateral preseptal cellulitis incited by local conjunctivitis caused by prescription eye drops. The patient was immunosuppressed, which allowed her local inflammation to progress to severe infection and, ultimately, to necrotizing fasciitis. This necessitated serial debridement by ophthalmology and otolaryngology teams and a prolonged course of intravenous antibiotics monitored by an infectious disease team. Despite these interventions, the patient’s vision did not return to baseline and she had persistent cosmetic and functional deformity. This case is unique due to the inciting incident of new prescription eye drops, the patient’s immunosuppressed state leading to severity of infection, and the severe bilateral disease burden.

## Introduction

Preseptal cellulitis is an infection of the eyelid and surrounding tissue anterior to the orbital septum. It is typically unilateral and bacterial in origin, often associated with sinonasal disease. In rare instances, it can extend to postseptal cellulitis or even lead to necrotizing fasciitis [1,2]. We present a rare case of bilateral preseptal necrotizing fasciitis with concurrent sinusitis, which was incited by conjunctival irritation secondary to prescription eye drops and exacerbated by the patient’s immunosuppressed state.

## Case presentation

A 58-year-old woman with a history of rheumatoid arthritis treated with tocilizumab, as well as depression treated with bupropion, presented to the emergency department with severe preseptal cellulitis and maxillary sinusitis. Her past ocular history was significant for uncomplicated cataract surgery in both eyes. She had been evaluated by her ophthalmologist one week prior to presentation, when she was prescribed brimonidine eye drops to treat persistently dilated pupils, thought to be a side effect of bupropion. Within one day she experienced rapidly progressive orbital swelling and pain, left greater than right. She also had increasing blurry vision, binocular diplopia, and upper respiratory symptoms, including sinonasal congestion and rhinorrhea. Her swelling was so significant that it caused obstruction of the visual axis secondary to eyelid edema. She presented to an outside hospital and was managed for a severe allergic reaction, where she received intravenous (IV) steroids and antihistamines. An outside CT scan was performed, which was concerning for extensive preseptal cellulitis and sinus disease. The patient was subsequently transferred to a tertiary care center for further evaluation and management.

The differential diagnosis for painful, bilateral eye swelling is broad but commonly includes viral and bacterial conjunctivitis, allergic conjunctivitis, blepharitis, trauma, contact dermatitis, herpetic dermatitis, preseptal cellulitis, angioedema, eczema, and autoimmune diseases.

**Figure 1:**
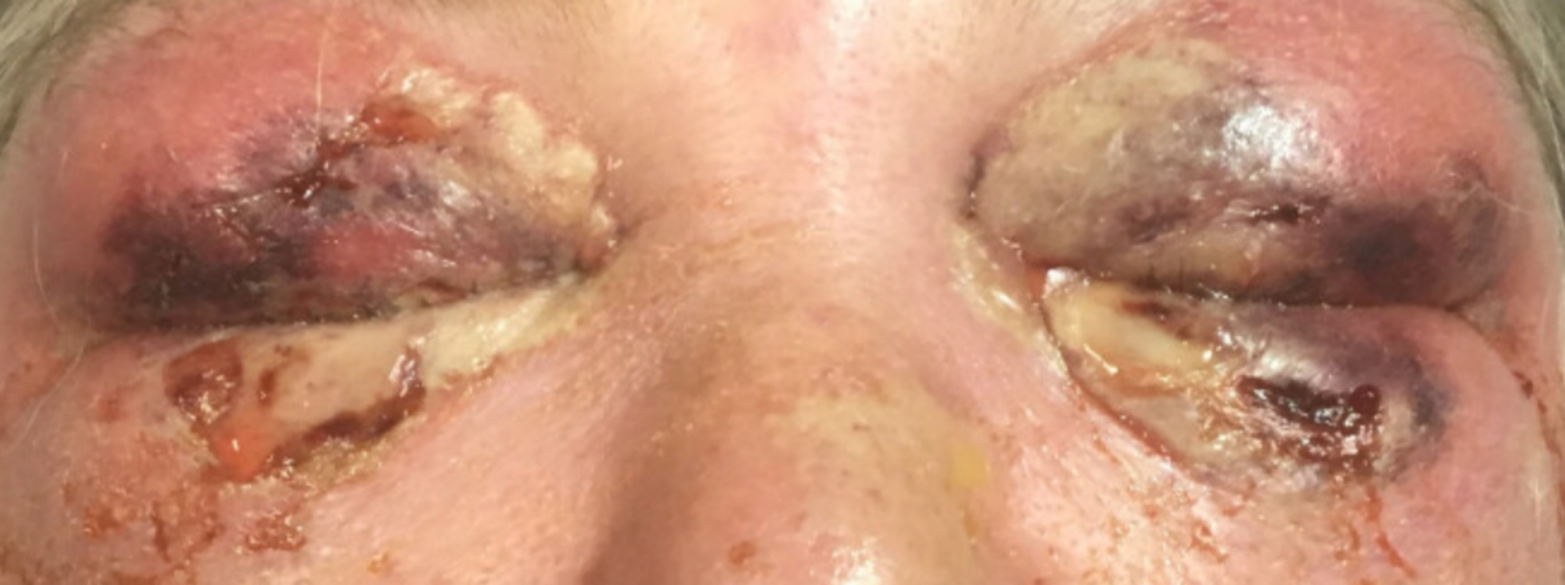
Patient's exam on initial presentation to tertiary care facility

On exam, the patient had erythema and swelling extending from the nose to forehead and laterally past the orbits bilaterally. Blackened, necrotic eyelids with skin breakdown and serosanginous discharge were present. There was tenderness of the malar prominence and neck bilaterally, without crepitus. The patient demonstrated significant lid edema, induration, ecchymosis, and tenderness; the left upper and lower lids were severely taught, and she had mild proptosis which was greater on the left. There was prominent conjunctival chemosis bilaterally. A 4 x 5 mm epithelial defect was present in the left inferior lid (Figure 1). Dilated fundus examination was within normal limits. Vision was measured with near card as 20/30 in the right eye and 20/400 in the left eye. The intraocular pressures were measured with tonopen at 19 mmHg in the right and 23 mmHg in the left. There was no afferent pupillary defect present, and extraocular motility was full without pain. The orbit was not noted to be tense or resistant to retropulsion.

Nasal endoscopic exam was performed without anesthetic due to suspicion for invasive fungal sinusitis in the setting of medical immunosuppression (given the patient’s history of rheumatoid arthritis treated with tocilizumab). This showed significant leftward septal deviation and universally friable mucosa that was fully sensate with no signs of necrosis. She had mucopurulent discharge at the middle meatus bilaterally and the right sphenoethmoid recess.

Relevant labs included a white blood cell count of 12.9 x 10^3^/mcL and C-reactive protein of 87.0 mg/L. Erythrocyte sedimentation rate (ESR) was normal.

**Figure 2:**
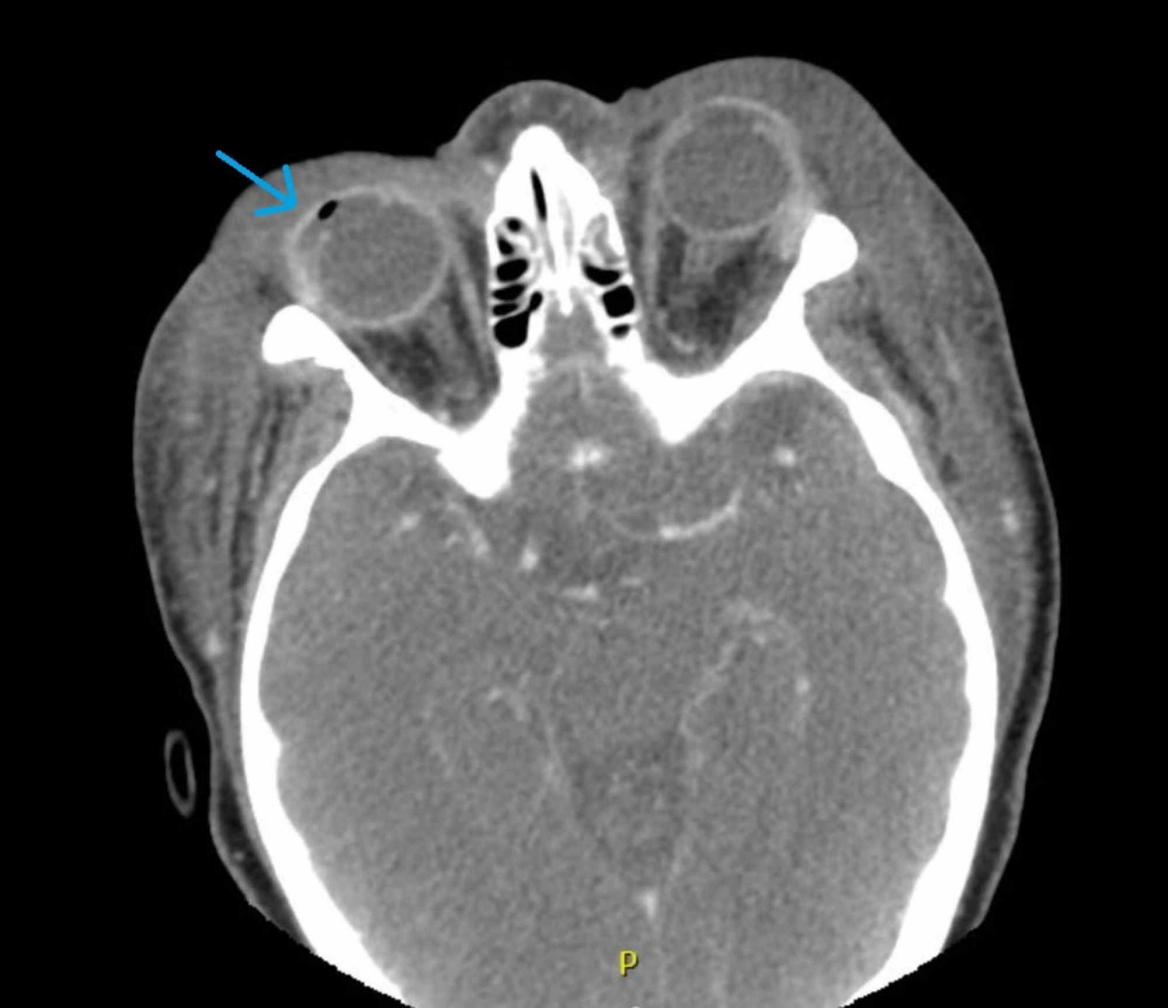
Axial CT imaging shows bilateral periorbital inflammation with right-sided abscess and emphysema

A maxillofacial CT scan with contrast demonstrated extensive periorbital soft tissue swelling with sinus inflammation and impaction of the bilateral maxillary and ethmoid sinuses and the left sphenoid sinus. She had bilateral conjunctival enhancement, with a rim-enhancing collection along the temporal right conjunctiva. This rim-enhancing fluid collection, indicative of an abscess, measured 5 mm x 13 mm with an intralesional focus of air (Figure 2). There was no postseptal stranding and no subperiosteal abscess identified. There was also bilateral cervical lymphadenopathy on neck CT. Magnetic resonance venography was obtained, which showed no involvement of the cavernous sinus.

**Figure 3:**
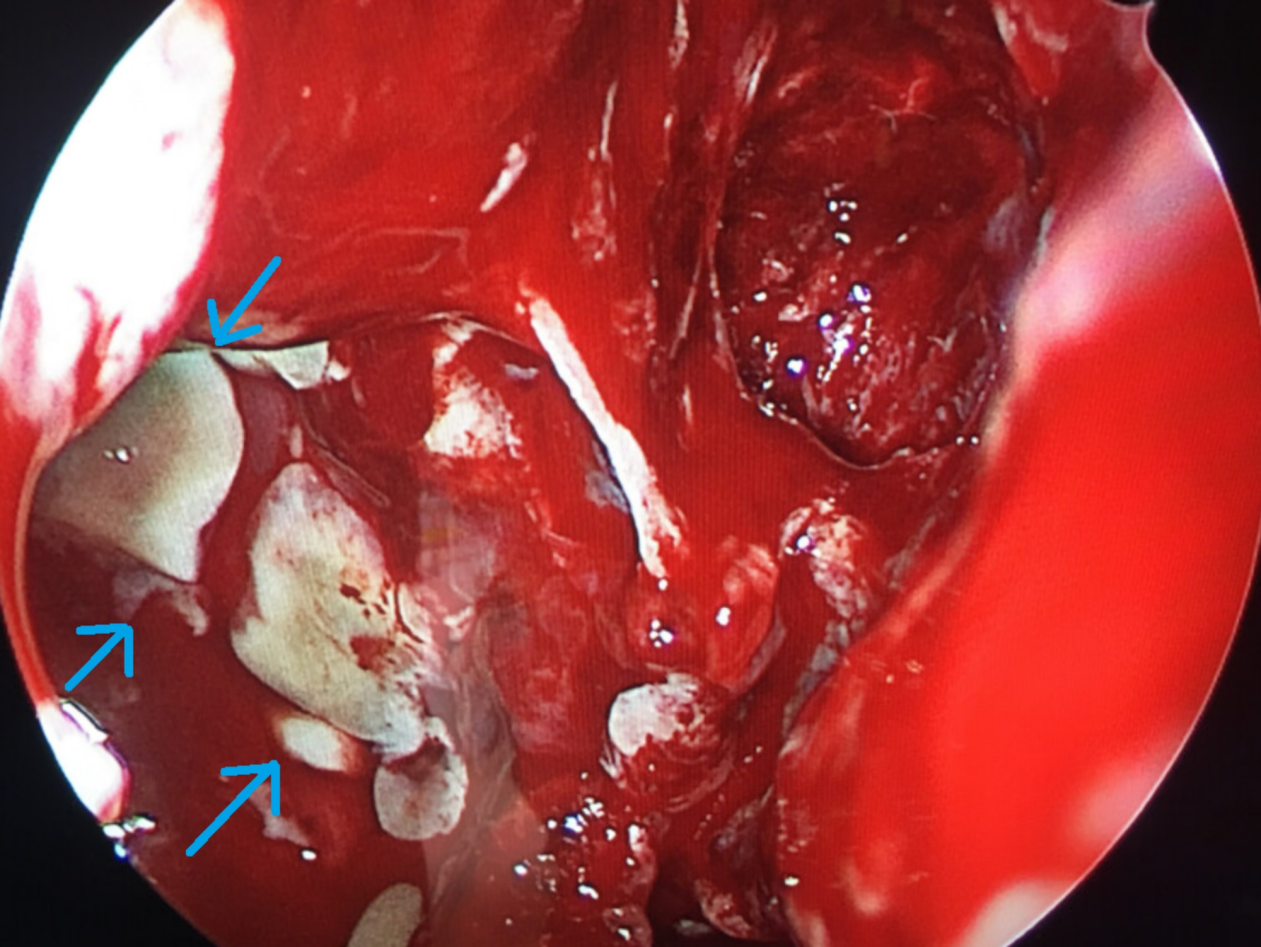
Endonasal findings of inflammation with acute infection denoted by purulence in the right maxillary sinus after sphenoethmoidectomy; there is no discrete necrosis to indicate invasive fungal disease

**Figure 4:**
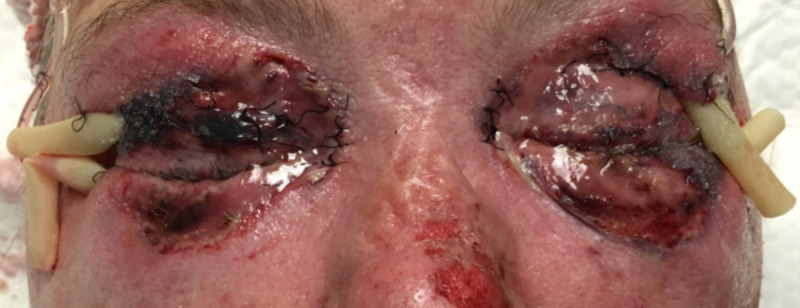
Postoperative photo following patient’s initial endonasal debridement and orbitotomy with washout

The patient was emergently taken to the operating room jointly by the ophthalmology and otolaryngology teams. Ophthalmology performed bilateral orbitotomy and washout. The orbital septum was noted to have a necrotic appearance, and there was “dishwater-appearing” darkened fluid in the preseptal space. Otolaryngology performed a functional endoscopic sinus surgery (FESS). Endonasal findings included diffuse inflammatory changes and purulence from bilateral maxillary and ethmoid sinuses; there was no evidence of devitalized tissue or necrosis, and frozen sections were negative for invasive fungal disease (Figure 3). The ophthalmology team placed penrose drains bilaterally for continued drainage (Figure 4).

**Figure 5:**
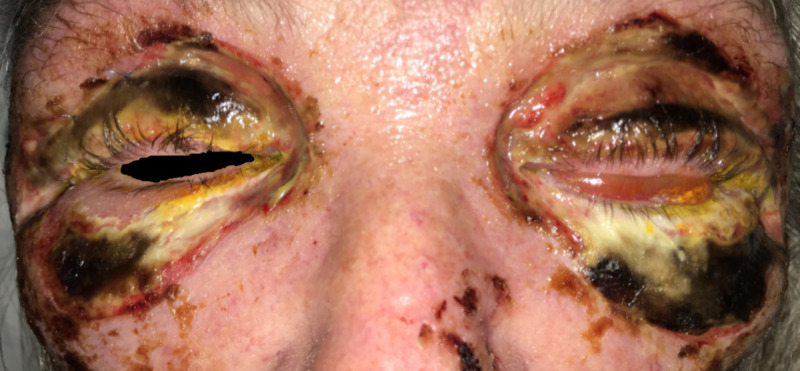
Findings after undergoing a second two-team debridement with ophthalmology and otolaryngology, with mild interval improvement and removal of drains

Intraoperative orbital pathology specimens were obtained, which showed coagulative necrosis and fibrinopurulent debris. Orbital cultures finalized with growth of Streptococcus pyogenes and methicillin-resistant Staphylococcus aureus (MRSA). Endonasal cultures grew only Streptococcus pyogenes. She was taken back to the operating room on hospital day 3 for a repeat orbital washout by ophthalmology and nasal debridement by otolaryngology, at which point the penrose drains were removed (Figure 5). She was also treated with four weeks of IV penicillin and vancomycin, IV steroids, and ophthalmic bacitracin ointment. Aggressive nasal hygiene was initiated, with saline irrigations and sprays, as well as fluticasone and oxymetazoline sprays. She also underwent temporary left tarsorrhaphy to protect the ocular surface.

**Figure 6:**
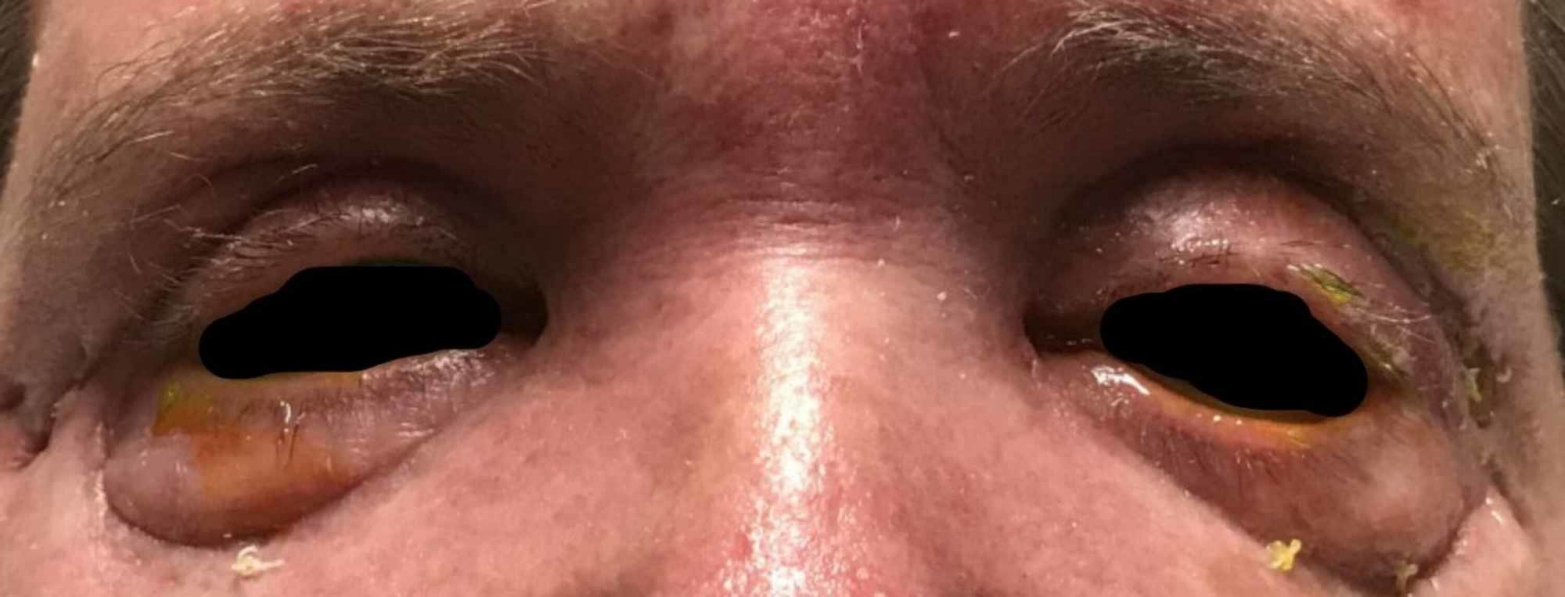
Long-term complications of patient’s infection include ectropion and lagophthalmos

After completion of therapy, she was noted to have persistent left lower lid cicatricial ectropion, foreign body sensation, and lagophthalmos (Figure 6). Her best corrected visual acuity three months after presentation was 20/25 in the right eye and 20/60 in the left eye. She continued a nasal regimen and a follow-up nasal endoscopy demonstrated expected post-FESS changes with patent sinuses bilaterally and no evidence of sinonasal disease.

## Discussion

Preseptal cellulitis is an infection of the eyelids and orbit anterior to the orbital septum. It is more commonly found in pediatric populations, in winter months, and in patients with upper respiratory infections. It is usually unilateral, and bilateral cases are typically associated with a prior infection or eye trauma. One study showed up that 78% of cases of orbital infections were directly caused by sinusitis, while other estimates say that up to 98% of cases of preseptal cellulitis are associated with concurrent rhinosinusitis, particularly disease involving the ethmoid sinuses [3,4]. It is hypothesized that this is due to direct spread of infection, or to venous drainage of the sinuses via ophthalmic veins. Certain congenital abnormalities, such as dehiscence of the lamina papyracea, can facilitate direct spread of infection. Less commonly, infection can be caused by iatrogenic or accidental trauma to the eye, or spread of infection from the ear, mastoid, or skin.

Most cases of preseptal cellulitis resolve completely with oral antibiotic therapy. However, risk factors for postseptal extension and complications include the presence of dental abscesses and an immunocompromised state [2]. One study of immunocompromised cancer patients with periorbital cellulitis demonstrated that these patients have higher rates of complication, morbidity, and all-cause mortality [5]. Cases that are refractory to oral antibiotics require hospital admission and IV antibiotics, and severe cases often necessitate surgical debridement endonasally by otolaryngology, with or without orbital washout by ophthalmology.

Common microbes associated with preseptal cellulitis include MRSA and Streptococcus pyogenes. In countries where vaccines are not readily available, Haemophilus influenzae and Streptococcus pneumoniae are common organisms involved [6]. The infiltration of bacteria in this patient included strep pyogenes, which also grew from her endonasal cultures and implicates sinonasal involvement. However, MRSA grew exclusively in ocular cultures. We hypothesized that this occurred as an extension of colonized skin MRSA that was facilitated by her previously injured conjunctiva. This infection necessitated a prolonged course of IV antibiotics.

Preseptal cellulitis is distinct from postseptal cellulitis, which is an infection of the orbital contents posterior to the septum but not including the globe itself. The Chandler system is often used to distinguish and classify these infections (Table 1) [7]. Exam findings more consistent with postseptal cellulitis include proptosis and restricted ocular motility with pain on attempted eye movement. Signs of optic neuropathy such as afferent pupillary defect or decreased color vision may be present in severe cases [8]. The workup of periorbital cellulitis should include a CT of the sinuses and orbits to differentiate preseptal and postseptal involvement, as well as evaluate for the formation of abscesses or other complications.

**Table 1:** Chandler classification system for periorbital cellulitis

Group	Description	Signs and symptoms	Treatment
1	Preseptal cellulitis	Unilateral eyelid edema, erythema, fever, tenderness	Antibiotics, sinonasal regimen (decongestants, mucolytics, irrigations); may consider oral antibiotics in mild cases
2	Orbital (postseptal) cellulitis	Chemosis, proptosis; +/- visual changes, afferent pupillary effect, ophthalmoplegia	IV antibiotics, aggressive sinonasal regimen; may consider endoscopic sinus surgery
3	Subperiosteal abscess	Chemosis; +/- proptosis, ophthalmoplegia, visual impairment	Urgent surgical decompression, IV antibiotics; may consider trial of medical therapy for smaller abscesses
4	Orbital abscess	Proptosis, chemosis, ophthalmoplegia, visual impairment	Urgent surgical decompression, IV antibiotics
5	Cavernous sinus thrombosis	Any of the above; +/- spiking fevers, toxemia, involvement of contralateral eye, papilledema	High-dose IV antibiotics that cross blood-brain barrier, bed rest, sinus surgery, +/- anticoagulation

One rare but deadly complication of periorbital cellulitis is necrotizing fasciitis. This is a severe and rapidly progressing infection of soft tissue with necrosis of the skin, subcutaneum, and fascia, and carries an overall high mortality rate of 25%-30% [9]. Ocular necrotizing fasciitis is typically unilateral and results from a preexisting cellulitis, with a reported mortality rate of 8%-15% [9]. Most cases of ocular necrotizing fasciitis in the literature were caused by recent ocular surgery or periorbital trauma. Necrotizing fasciitis most commonly occurs in patients with diabetes mellitus or other immunocompromised states [10]. This patient was on a prescribed injectable biologic agent for her rheumatoid arthritis, but was not diabetic; it is likely that her immunosuppression contributed to the severity of her infection. Additionally, she received steroids without antibiosis at the outside hospital, which may have propagated her infection.

Necrotizing fasciitis can be serious, possibly leading to septicemia and death [11]. Treatment necessitates serial surgical debridement and broad-spectrum antibiotics, and some research has shown benefit to hyperbaric oxygen therapy [12,13]. A delay in surgical debridement is the highest risk factor for all-cause mortality in necrotizing fasciitis [10]. Yet despite appropriate early interventions, prolonged morbidity is common and many patients require multiple surgical procedures that result in disfigurement [14]. Ocular necrotizing fasciitis in particular can result in serious cosmetic deformities such as ectropion, which can also lead to dry eye and corneal abrasion [15].

This case is particularly unique for many reasons. The patient had recently been prescribed buproprion for depressive symptoms, a rare side effect of which is pupillary dilatation. To treat this, she was prescribed brimonidine drops by her ophthalmologist and had a local reaction to the drops with inflammation of the conjunctiva and nearby tissues. There are previously described cases of periorbital cellulitis caused by irritated conjunctiva. It is theorized that the patient’s reaction to her eyedrops contributed to the spread of sinonasal infection and skin flora causing preseptal cellulitis, and that her immunosuppressed state led to the severity of her infection and necrotizing fasciitis. Because of this she required aggressive treatment that included multiple trips to the operating room for nasal and orbital debridement.

## Conclusions

Preseptal cellulitis is an infection of the soft tissues anterior to the orbital septum. These types of infections are often unilateral and attributed to extension of sinonasal disease. Our case was unique in that it included local irritation and conjunctivitis caused by new prescription eye drops that may have facilitated spread of infection, and her immunosuppressed state led to a severe, bilateral necrotizing fasciitis. This reminds us that it is important to be judicious in prescribing all new medications, including seemingly innocuous medications like buproprion and brimonidine. This is particularly important in immunosuppressed patients, in whom even mild allergic conjunctivitis should be managed vigilantly for associated complications. It is imperative that patients with preseptal cellulitis receive comprehensive workup to monitor for the severe complications that can occur. This case serves as an excellent reminder that these infections can rapidly progress to life-threatening necrotizing fasciitis, and often require attentive co-management between ophthalmology, otolaryngology, and medicine teams.
